# Collectivism, individualism and COVID-19 prevention: a cross sectional study of personality, culture and behavior among Canadians

**DOI:** 10.1080/21642850.2022.2069571

**Published:** 2022-04-30

**Authors:** Kiffer G. Card

**Affiliations:** a Faculty of Health Sciences, Simon Fraser University, 8888 University Drive, Burnaby, BC, Canada; b The GenWell Project Society, Toronto, Ont., Canada

**Keywords:** Collectivism-individualism, cultural orientations, personality, agreeableness, COVID-19, mask-wearing, social distancing

## Abstract

**Background:**

Collectivism has been identified as a protective factor against COVID-19 – perhaps due to increased conformity with social norms regarding prevention behaviors. Other studies have also found that individualism can inspire uptake of preventative behaviors as a means of personal protection. It is possible that these cultural orientations may promote different patterns of prevention (e.g. mask wearing vs. social distancing). Furthermore, existing studies examining the role of individualism and collectivism during the COVID-19 pandemic have frequently failed to account for other psychological processes, including differences in personality, which could help provide a better understanding of the psychological process underlying prevention behavior.

**Methods:**

Participants were recruited using social media advertisements. The Cultural Orientations Scale measured individualism–collectivism and hierarchism-egalitarianism. The Ten Item Personality Inventory measured the five factor model of personality. Multivariable models, dominance analyses and structural equation mediation tests were used to identify the most important predictors of COVID-19 prevention behavior (i.e. mask-wearing, hand-washing, reducing social interactions, physical distancing, staying at home and social bubbling), controlling for demographic and situational factors.

**Results:**

Among 774 participants, most (i.e. 60–80%) reported uptake of COVID-19 prevention behaviors. Higher vertical (hierarchical) collectivism was associated with staying at home and higher horizontal (egalitarian) individualism was associated with mask-wearing and reducing social interactions. Neither Vertical Collectivism nor Horizontal Collectivism were significantly associated with any of the prevention behaviors when controlling for personality traits and confounding variables. Agreeableness was identified as a key mediator of the correlation between these cultural orientations on general uptake of COVID-19 prevention behaviors.

**Conclusions:**

Cultural orientations (e.g. collectivism-individualism, hierarchism-egalitarianism) and personality traits (e.g. Agreeableness) are salient correlates of COVID-19 prevention behaviors and therefore should be accounted for in the development, design and delivery of health promotion messages aiming to increase uptake of these behaviors.

## Introduction

### The relationship between infectious disease and culture

Disease causing pathogens, such as SARS-COV-2, are nothing new to human societies (Piret & Boivin, 2021). In fact, many scientists and historians believe that some of the most important features of human culture were shaped by our past interactions with microbes and our (perhaps unconscious) desire to evade disease (Schaller & Murray, 2010). The historical impact of pathogens on human morbidity and mortality has undoubtedly given rise to considerable selective evolutionary pressures, which could, in theory, shape our psychology and culture (Fumagalli et al., 2011). These pressures are hypothesized to have given rise to a ‘behavioural immune system’ (Schaller & Duncan, 2007). One such cultural dimension that may have arisen as part of this immune system is cultural collectivism (Thornhill & Fincher, 2014). Collectivism describes a cultural disposition towards prosocial behavior and group-based self-conceptualization. Along with its dipole, individualism, it is thought of as one of the most important dimensions distinguishing cultures around the globe (Heine & Ruby, 2010; Markus & Kitayama, 1991; Triandis, 2001).

Supporting the idea that disease causing pathogens serve as a selective pressure for collectivism, people (and cultures) who are collectivistic tend to identify more readily with their groups, comply more strictly to group norms and are wearier of outside threats – whether human or otherwise (Douglas, 2003; Triandis, 2001). The tight-knit, closed-off features of these societies is therefore hypothesized to limit the acquisition of new pathogens into one’s group; while stronger uptake of group norms could support the emergence of cultural purity rituals that could also reduce pathogenic exposure (Douglas, 2003). Empirically, researchers have demonstrated that the regional prevalence of disease-causing pathogens is in fact correlated with higher average collectivism and lower individualism (Cashdan & Steele, 2013; Fincher, Thornhill, Murray, & Schaller, 2008; Morand & Walther, 2018). This may support the theory that collectivism offers some competitive advantage against disease-causing pathogens – and therefore collectivism has emerged more strongly in areas with higher rates of disease (Fincher et al., 2008).

## The role of individualism–collectivism in the COVID-19 pandemic

Evidence from the black plague and the COVID-19 pandemic further press the case that microscopic forces can and do, have monumental cultural and social impacts (Patterson, McIntyre, Clough, & Rushton, 2021). When looking at this empirical evidence, several studies conducted in Asian countries during the COVID-19 pandemic suggest that collectivism may increase as a cultural response to infectious disease (Han, Ren, Wu, Liu, & Zhu, 2021; Na et al., 2021). Further, cross-country comparisons show that greater collectivism is associated with better COVID-19 related outcomes (Cao, Li, & Liu, 2020; Jiang, Wei, & Zhang, 2021; Maaravi, Levy, Gur, Confino, & Segal, 2021; Rajkumar, 2021; Webster, Howell, Losee, Mahar, & Wongsomboon, 2021) – perhaps due to the relatively rapid and unified responses that collectivistic countries can mount relative to their individualistic counterparts (Chen et al., 2021a, 2021b). Collectivism was even cited among lay members of the public in qualitative research examining the prevention behaviors of Chinese–Canadians during the early wave of the COVID-19 pandemic (Lee et al., 2021) – giving face validity to the hypothesized link between collectivism and prevention behavior. These are further supported with individual-level quantitative research showing that greater collectivism is associated with more support for and uptake of COVID-19 prevention behaviors (Bok, Shum, Harvie, & Lee, 2021; Cho, Guo, & Torelli, 2022; Lu, Jin, & English, 2021; Travaglino & Moon, 2021; Yu, Lau, & Lau, 2021). Greater collectivism has also been shown to be correlated with fear of COVID-19 (Ahuja, Banerjee, Chaudhary, & Gidwani, 2021; Germani, Buratta, Delvecchio, & Mazzeschi, 2020). For example, Schneider and colleagues reported that the level of individualism–collectivism an individual reported was the *most important predictor of risk perception* – even higher than regional COVID-19 case counts (Schneider et al., 2021). Taken together this evidence certainly speaks strongly to a dynamic relationship between culture and disease causing pathogens (Douglas & Calvez, 1990).

## The role of personality in the COVID-19 pandemic

Naturally, not all studies looking at the relationship between prevention behavior and collectivism lend support to the narrative outlined above. Several studies have found that individualism *also* contributes to the uptake of COVID-19 prevention behaviors (Galang, Johnson, & Obhi, 2021; Miyajima & Murakami, 2021; Mo & Park, 2021; Shekriladze, Javakhishvili, & Chkhaidze, 2021; Xiao, 2021) – after all falling ill to COVID-19 is not usually in one’s self-interest. This raises questions about the role that culture plays in influencing individual behavioral responses to COVID-19. Indeed, if both individualism *and* collectivism predict COVID-19 prevention behavior, why would one assume that these, and not other confounding psychological processes, are the drivers of COVID-19 prevention behaviors? For example, a growing body of literature is showing that other individual level psychological factors – such as those from the five-factor model of personality – are associated with COVID-19 perceptions and prevention behaviors (Wright & Fancourt, 2021). For example, people with lower emotional stability appear to be more concerned with the pandemic but have also had the most negative experiences during this period – which might motivate coping behaviors that reduce prevention uptake (Anglim & Horwood, 2021; Fink et al., 2021; Iterbeke & De Witte, 2021; Modersitzki, Phan, Kuper, & Rauthmann, 2021; Pilch, Wardawy, Probierz, & Lahiri, 2021; Troisi, Nanni, Riconi, Carola, & Di Cave, 2021; Zettler et al., 2022). Meanwhile, people who are more agreeable or conscientiousness tend to be more willing to comply to COVID-19 prevention guidelines (Agbaria & Mokh, 2021; AL-Omiri et al., 2021; Gollwitzer, Platzer, Göritz, Zwarg, & Twardawski, 2021; Gori, Topino, Palazzeschi, Di Fabio, & Topa, 2021; Han, 2021, 2021, 2021; Kanazawa, 2021; Kohút, Kohútová, & Halama, 2021; Krupić, Žuro, & Krupić, 2021; Martinsen, Furnham, Grover, Arnulf, & Horne, 2021; Rammstedt, Lechner, & Weiß, 2022; Starcevic & Janca, 2022). Unfortunately, studies examining the relationship between culture and COVID-19 prevention behaviors have not often accounted for these factors. The seeming importance of the big five personality traits should rouse interest among researchers investigating the role of culture and collectivism on COVID-19 prevention. Not only can factors such as agreeableness, extraversion and openness map onto the concepts of collectivism and individualism, but individualism and collectivism can also be thought of as internalized psycho-cultural orientations or even personality traits themselves (Laher & Dockrat, 2019). It, thus, makes sense that they should be examined in tandem.

## Nuanced models of cultural orientation

Furthermore, it may be useful to adopt a more nuanced model of individualism and collectivism in order to describe the seemingly contradictory findings regarding collectivism and individualism and the complex cultural motives that might underlie these relationships. Fortunately, previous efforts have been undertaken to offer more nuanced models of individualism and collectivism (Singelis, Triandis, Bhawuk, & Gelfand, 1995; Triandis, 2001). Such models highlight multiple types of individualism and collectivism that are important to defining cultural orientation and have proven useful in studying the COVID-19 pandemic response. In particular, the additional dimensions of *vertical hierarchy* and *horizontal egalitarianism* have been integrated with collectivism-individualism to provide a parsimonious, yet nuanced, model of cultural orientation (Singelis et al., 1995; Triandis & Gelfand, 1998). This model adds nuance to traditional definitions of individualism and collectivism by differentiating between *vertical* and *horizontal* subtypes of collectivism and individualism (Fatehi, Priestley, & Taasoobshirazi, 2020; Triandis & Gelfand, 1998). According to Singelis et al. (1995), ***Vertical Orientations*** theoretically relate to the perception that social relationships are inherently hierarchical, that roles are differentiated, and that inequalities between individuals are fundamentally unavoidable. Individualistic oriented individuals are believed to respond to this perception by jockeying for position by navigating and rising through the social hierarchy; while collectivistic oriented individuals are believed to conform or submit to their place in the social order. ***Horizontal Orientations***, on the other hand, theoretically relate to the perception that individuals are all essentially the same and equal. The importance of social position, authority and power is diminished within social orientations. Individualists with a horizontal orientation, therefore, give little thought to others – they do their own thing; while collectivists with a horizontal orientation approach social relationships more collaboratively – feeling equal with those in their group.

These nuanced dimensions of vertical and horizontal individualism–collectivism have important implications for how culture may influence COVID-19 prevention. For example, vertical hierarchism may produce greater obedience and horizontal egalitarianism greater empathy for others (Atalay & Solmazer, 2021; Leonhardt & Pezzuti, 2022) – leading to greater uptake of COVID-19 prevention behaviors, regardless of their individualism and collectivism. Indeed, while collectivism may be generally predictive of better uptake of prevention behaviors, egalitarian individualists might also be inclined to comply with public health guidance, even if it requires some self-sacrifice. Further, hierarchical cultural beliefs that emphasize vertical social differences might be especially motivating for collectivistic individuals. The moderating role of cultural hierarchism-egalitarianism (i.e. the extent to which individual roles and statuses are equal) thus provides added depth to those made when only considering collectivism-individualism (i.e. extent to which somebody feels integrated in a social group).

## Cultural orientations and the COVID-19 pandemic

Empirical applications of the integrated cultural orientation model support the value of accounting for the added complexity of hierarchism-egalitarianism. For example, Xiao (2021) demonstrated that both vertical collectivists and horizontal individualist were more willing to comply with pandemic prevention measures, while vertical individualism was associated with a lower willingness to comply (Xiao, 2021). Similar results were shown by Mo & Park when examining the positive effect of horizontal individualism and vertical collectivism on perceptions about mask-wearing, as well as the negative effect of vertical individualism (Mo & Park, 2021). This research highlights the importance of culture as a motivation for compliance. Underscoring this effect, recent research has shown that vertical collectivism plays an important moderating role in anxiety-related to COVID-19 by promoting a greater willingness to isolate with one’s family as well as raising concern about COVID-19 infection among close family members (Wu, Deng, & Liu, 2021). Atalay and Solmazer (2021) also highlighted the role of vertical cultural orientations in producing willingness to stay home (Atalay & Solmazer, 2021). In examining other motives related to cultural orientation, Travaglino and Moon (2021) showed that vertical collectivism was associated with shame and horizontal collectivism with trust in government – both lending themselves to greater compliance with COVID-19 (Travaglino & Moon, 2021). Their research, however, highlighted the important context-dependence of these measures across countries: necessitating country and context-specific evaluations.

## Study objectives

Given the evidence summarized above, it is clear that considering nuanced models of cultural orientations along with personality traits can help isolate the independent relationship of these on COVID-19 prevention; thereby assisting public health leaders in responding to COVID-19 (Bayeh, Yampolsky, & Ryder, 2021; Caulkins, 1999; Erman & Medeiros, 2021; Nair & Selvaraj, 2021; Siritzky, Condon, & Weston, 2022). This exploratory analyses may be especially beneficial when considering how public health messaging can be best tailored to promote widespread uptake among individuals with diverse personal characteristics and cultural dispositions (Borah, Hwang, & Hsu, 2021; Clark, Davila, Regis, & Kraus, 2020; Courtney, Felig, & Goldenberg, 2022; Kemmelmeier & Jami, 2021; Mo & Park, 2021; Yu et al., 2021). As such, the present exploratory study sought to examine the independent relationships of cultural orientations and personality traits with the uptake of COVID-19 prevention behaviors among people living in Canada.

## Methods

### Data collection

Participants were recruited using paid advertisements promoted on Facebook, Instagram, Twitter and Google. Advertisements ran between April 21st and June 1st 2021, during the Third Wave of the COVID-19 pandemic, when vaccinations were beginning to be widely available to the general public in Canada. Advertisements and surveys were available in French and English. Eligibility criteria restricted participation to those who were 16 years of age or older, lived in Canada, and provided informed consent. The survey took approximately 21-minutes (Q_1_–Q_3_: 10–35 minutes) and assessed (1) social connection, (2) health and wellbeing, (3) participant demographics, (4) psychological psychometrics, (5) health behavior and healthcare access and (6) workplace and built environments. To reduce participant burden, only a subset of participants completed each of the final three modules. Participants were compensated for completing the study through entry into a random lottery. Twenty-five prize winners received $100 gift card prizes. Ethics review for the CSCS was conducted by the Research Ethics Board at the University of Victoria. All participants provided informed consent prior to participation.

## Variables

Data for this analysis were drawn for modules 1, 2, 3 and 4 and measured participant’s COVID-19 Prevention Behaviors, their levels of Individualism and Collectivism, and their sociodemographic characteristics. Section 4 was one of the randomized modules, and thus not all participants were included in this study.

- ***COVID-19 Prevention Behaviors.*** Participants were asked to indicate whether they had been vaccinated (No, 1 dose, 2 doses) and the extent to which they had been practicing physically distancing themselves by 2 meters from others; wearing a mask in public; washing their hands often; reducing the number of people they were interacting with; avoiding non-essential trips in the community; socializing indoors only with people in their immediate household. Participants responded to each prompt using a 3-point Likert Scale (‘Not at all’, ‘Somewhat’, ‘Very Closely’).
- ***Individualism–Collectivism.*** The Cultural Orientations Scale (discussed above) was used to measure individualism and collectivism (Triandis & Gelfand, 1998). This scale distinguishes between vertical and horizontal subtypes of individualism and collectivism through the use of four subscales: The Vertical Individualism (VI) subscale measures competitiveness (e.g. ‘Winning is everything’). The Vertical Collectivism (VC) subscale measures duty and self-sacrifice (e.g. ‘It is my duty to take care of my family, even when I have to sacrifice what I want’). The Horizontal Individualism (HI) subscale measures self-reliance and independence (e.g. ‘I'd rather depend on myself than others’); The Horizontal Collectivism (HC) subscale measures pro-sociality (e.g. ‘I feel good when I cooperate with others’). Each of the COS subscales consists of four items, which are scored and summed separately. Each item is measured on a 9-point Likert scale, ranging from (1) ‘Never or Definitely No’ to (9) ‘Always or Definitely Yes.’ Thus, final scores on each subscale range from 4 to 36.
- ***Personality Traits.*** The big five personality characteristics were assessed using the Ten Item Personality Inventory (TIPI), which was designed as a short measure for the five factor model of personality. The TIPI measure has been shown to have acceptable reliability and convergence with other five factor model measures (Gosling, Rentfrow, & Swann, 2003). The TIPI consists of five sub-scales, one for each big five factor (i.e. openness to experience, conscientiousness, extraversion, agreeableness and neuroticism). Participants are introduced to the questions with the following text:Here are a number of personality traits that may or may not apply to you. Please indicate the extent to which you agree or disagree with that statement. You should rate the extent to which the pair of traits applies to you, even if one characteristic applies more strongly than the other.
  They are then presented with pairs of terms related to each sub-scale and asked to rate the extent to which they agree the terms represent them on a 7-point Likert scale from ‘Agree Strongly’ to ‘Disagree Strongly’ each subscale is scored as the average value of two items: one positively scored item and one reverse scored item. For example, the term pairs (a) ‘Extraverted, enthusiastic’ and (b) ‘Reserved, quiet’ are used to score the extraversion subscale (See Supplemental Table 2 for term pairs representing each factor). Each subscale score ranges from 2 to 14.
- ***Demographic and lifestyle characteristics.*** Demographic and lifestyle characteristics included age, gender (man, non-binary, woman), relationship status (single vs. in a relationship), sexual orientation identity (2SLGBTQ+ vs. heterosexual), ethnicity, educational attainment (High School Diploma or Lower, College or Advanced Skills Training, Bachelor’s Degree or Graduate/Professional Degree), student status (Current student vs. not), employment status (Employed vs. not), distance learning and working situation (All or most of the time, some of the time, A little or none of the time), income (Less than $30,000, $30,000 to $59,999, $60,000 to $89,999, $90,000 to $119,999, $120,000+) and province of residence (Alberta, British Columbia, Manitoban, New Brunswick, Newfoundland and Labrador, Nova Scotia, Ontario, Prince Edward Island, Quebec, Saskatchewan and The Territories). These factors were included to account for the demographic and lifestyle factors that may shape both COVID-19 prevention behaviors, cultural orientations and personality traits. These were also important to control for given the non-representative opt-in nature of the study (Haddad et al., 2022; Pforr & Dannwolf, 2017).

## Statistical analysis

All statistical analyses were conducted in R Studio. First, descriptive statistics were calculated for each variable of interest for the full analytical sample using the tableone package (Yoshida et al., 2021). Second, histograms, correlation plots, and Spearman correlation coefficients were created using base R and ggplot2 package to examine associations between Cultural Orientations Scale subscales (R Core Team, 2021; Wickham et al., 2021). Third, the suitability of the Cultural Orientations Scale was assessed by using the psych package to conduct Cronbach α calculations, exploratory factor analysis, and confirmatory factor analysis of the scales (See Supplemental Table 1; Revelle, 2021). Composite reliability estimates were also calculated for the COS subscales using the semTools package and reliability() function (Jorgensen et al., 2021). These analyses indicated that each measure had satisfactory internal consistency (i.e. ≥ 0.8), composite reliability (i.e. ≥ 0.80), and the original factor structure was nearing acceptability according to standard fit criteria for confirmatory models (CFI = 0.91; TLI = 0.89; RMSEA = 0.09 [0.09, 0.10, *p* = .000]; SRMR = 0.09). While there was some evidence to suggest the fit of the confirmatory model could have been improved by dropping some poor fitting items, the widespread use of the scale provided sufficient motivation to keep the scale as originally designed – thereby facilitating comparisons with other studies. Furthermore, dropping the poor fitting items reduced internal consistency. Similar analyses were not undertaken on the TIPI, as the factor structure and Cronbach’s alpha are known to be low for the scale, given that each factor consists of two items and that the items of each subscale capture different dipoles of the concept being measured. As such, internal consistency and factor structures are well known to be misleading for these types of scales (Gosling, 2021, 2003; Kline, 2000; Woods & Hampson, 2005). Fourth, multivariable binary logistic regression models, created using the base glm() function, tested the association between each subscale of the Cultural Orientations Scale (i.e. HC, VC, HI and CI) and each of the COVID-19 prevention behaviors (i.e. Vaccination, Handwashing, Mask Wearing, Reduced Interactions, Physical Distancing and Staying at Home). The vaccine uptake model compared people who were fully or partially vaccinated to those who were not vaccinated (referent). This referent group was selected as partial vaccination likely reflects an intent to be fully vaccinated, but due to the timing of the survey, not everyone was eligible to be fully vaccinated. The other models compared people who were ‘very closely’ adhering to those who were ‘somewhat’ or ‘not at all’ adhering to the guideline. These referent groups and categories were selected because strict adherence to the prevention strategies provided the greatest indication of cultural support – whereas individuals might adhere ‘sometimes’ due to factors beyond their control (i.e. mandates for entry into public places). Each model controlled for confounding demographic and lifestyle factors (e.g. work from home). Further, with the exception of the model for vaccine uptake, each model also controlled for confounding by vaccination status (as qualitative community consultations indicated that vaccination status would be an important factor in other COVID-19 factors).

Next, and fifth, an index score measuring general uptake of COVID-19 prevention behaviors was created. While an index is a crude sum score measure, it helps capture an overall rigidity to COVID-19 prevention behaviors. Each of the outcomes, with the exception of vaccination status, were scored from 0 to 2. ‘Very closely’ adhering to a guideline was scored as 2 points, ‘somewhat’ adhering to a guideline was scores as 1 point, and adhering ‘not at all’ was scored as 0 points. Final scores ranged from 0 to 12, with higher scores indicating greater uptake. Cronbach’s α was calculated for this measure as a measure of internal consistency (α = 0.80). Exploratory factor analysis was used to examine the multivariate relationships of the prevention behaviors. As shown in Table 1, these revealed a personal hygiene factor and a social distancing factor. The sub-indices and overall index were analyzed using dominance analysis (Azen & Traxel, 2009; Budescu, 1993) to identify the most important variables in predicting gender uptake of COVID-19 prevention behaviors. Dominance analyses, conducted using the dominanceanalysis package (Navarrete & Soares, 2020), assessed the most important factors, by their average conditional contribution to the model R-squared value. Conditional dominance figures were constructed to assess the relative importance of explanatory factors to prevention practice uptake. These model show the main factors of interest. All confounding relationships and main relationships of interest were considered, but the confounders were held constant.

**Table 1.** Factor analysis of COVID-19 prevention behavior index.

	1 Factor	2 Factors	
		Social distance	Personal hygiene
Cronbach’s α	0.80	0.76	0.58
Item factor loadings			
Wash your hands often	0.539	0.237	0.625
Wear a mask in public	0.646	0.444	0.488
Physically distance yourself by 2 meters from others	0.679	0.551	0.390
Avoid non-essential trips in the community	0.654	0.666	0.224
Socialize indoors only with people in your immediate household	0.566	0.515	0.255
Reduce the number of people you interact with	0.744	0.614	0.408

Based on the results of these dominance analyses, the sixth step undertaken was to test the mediating relationships for notably important personality traits to examine whether they mediated the relationship between cultural orientations and COVID-19 Prevention Behavior Index scores. Mediation analyses were conducted using structural equation models programed using the lavaan package, which allowed us to calculate the direct and indirect effects for each variable of interest (Rosseel et al., 2022). Models controlled for each demographic variable, and the model simultaneously tested the effects of all cultural orientation measures and personality traits at once. The significance of the effects were tested using 1000 bootstrapped samples and 95% confidence intervals were computed (Tingley, Yamamoto, Hirose, Keele, & Imai, 2014). Regression estimates and *p*-values were graphed and presented in a path model.

## Results

A total of 2,286 valid responses were collected. Of these, 1,071 completed the relevant modules necessary for inclusion into this exploratory study. Table 2 shows descriptive statistics for the final analytic sample, which consisted of 774 participants due to missing data across other variables. The final sample was reasonably diverse and the median age of the sample was 38.35 and approximately half male (44.8%) and half female (53.7%). Most participants were straight (65.4%) single (52.3%), had incomes below $60,000 CAD (68.6%), and identified as white (66.4%).

**Table 2.** Descriptive characteristics for analytic sample.

Variable	Statistic
*Age (Numeric–Mean [SD])*	38.35 (15.07)
*Gender (N [%])*	
Man	347 (44.8)
Non-binary	11 (1.4)
Woman	416 (53.7)
*Sexual Orientation (N [%])*	
Straight	506 (65.4)
2SLGBTQ+	268 (34.6)
*Relationship Status (N [%])*	
In a relationship	369 (47.7)
Single	405 (52.3)
*Ethnicity (N [%])*	
African, Caribbean or Black	75 (9.7)
Arab	16 (2.1)
Chinese	23 (3.0)
Filipino	6 (0.8)
Indigenous	55 (7.1)
Japanese	3 (0.4)
Korean	3 (0.4)
Latin American	29 (3.7)
South Asian	15 (1.9)
Southeast Asian	5 (0.6)
West Asian	5 (0.6)
White	514 (66.4)
Other	25 (3.2)
*Household Income (N [%])*	
Less than $30,000	221 (28.6)
$30,000 to $59,999	232 (30.0)
$60,000 to $89,999	125 (16.1)
$90,000 to $119,999	99 (12.8)
$120,000 +	97 (12.5)
*Employment Status (N [%])*	
Unemployed	142 (18.3)
Employed	632 (81.7)
*Working From Home (N [%])*	
All or mostly	691 (89.3)
Some of the time	57 (7.4)
Very little or not at all	26 (3.4)
*Educational Attainment (N [%])*	
High School Diploma or Lower	113 (14.6)
College or Advanced Skills Training	289 (37.3)
Bachelor’s Degree	151 (19.5)
Post-Graduate/Professional Degree	221 (28.6)
*Current Student (N [%])*	
No	618 (79.8)
Yes	156 (20.2)
*Online Learning (N [%])*	
All or mostly	691 (89.3)
Some of the time	57 (7.4)
Very little or not at all	26 (3.4)
*Province of Residence (N [%])*	
Alberta	103 (13.3)
British Columbia	191 (24.7)
Manitoba	43 (5.6)
New Brunswick	35 (4.5)
Newfoundland and Labrador	29 (3.7)
Nova Scotia	30 (3.9)
Ontario	164 (21.2)
Prince Edward Island	14 (1.8)
Quebec	94 (12.1)
Saskatchewan	22 (2.8)
The Territories	49 (6.3)
*Adherence to COVID-19 Prevention Guidelines*	
*Prevent Uptake Index Score–Overall (N [%])*	9.63 (2.51)
*Prevent Uptake Index Score–Personal Hygiene (N [%])*	3.43 (0.86)
*Prevent Uptake Index Score–Social Distance (N [%])*	6.21 (1.88)
*Vaccination (N [%])*	
Not Vaccinated	168 (21.7)
Partially Vaccinated (1 dose)	439 (56.7)
Fully Vaccinated (2 doses)	167 (21.6)
*Wash your hands often (N [%])*	
Not at All	25 (3.2)
Somewhat	164 (21.2)
Very Closely	585 (75.6)
*Wear a mask in public (N [%])*	
Not at All	30 (3.9)
Somewhat	170 (22.0)
Very Closely	574 (74.2)
*Physically distance yourself by 2 meters from others (N [%])*	
Not at All	45 (5.8)
Somewhat	285 (36.8)
Very Closely	444 (57.4)
*Avoid non-essential trips in the community (N [%])*	
Not at All	43 (5.6)
Somewhat	242 (31.3)
Very Closely	489 (63.2)
*Socialize indoors only with people in your immediate household (N [%])*	
Not at All	54 (7.0)
Somewhat	250 (32.3)
Very Closely	470 (60.7)
*Reduce the number of people you interact with (N [%])*	
Not at All	46 (5.9)
Somewhat	235 (30.4)
Very Closely	493 (63.7)
*Cultural Orientations Scale Subscale Scores*	
Horizontal Individualism *(Numeric–Mean [SD])*	25.33 (6.93)
Vertical Individualism *(Numeric–Mean [SD])*	20.01 (7.34)
Horizontal Collectivism *(Numeric–Mean [SD])*	24.93 (6.60)
Vertical Collectivism *(Numeric–Mean [SD])*	24.17 (6.88)
*Ten Item Personality Inventory Scale Subscale Scores*	
Extraversion *(Numeric–Mean [SD])*	7.85 (2.47)
Agreeableness *(Numeric–Mean [SD])*	9.64 (2.37)
Conscientiousness *(Numeric–Mean [SD])*	9.59 (2.60)
Emotional Stability *(Numeric–Mean [SD])*	8.73 (2.62)
Openness to Experience *(Numeric–Mean [SD])*	9.21 (2.36)

Among 774 participants, most were fully or partially vaccinated (79.1%) and most ‘very closely’ adhered to guidelines for hand-washing (74.5%), mask wearing (73.4%), staying home (62.5%), reducing social contacts (62.2%) and socializing in-person only with household members (60.4%). For the four cultural orientations, relatively high median scores were observed for HI, HC and VC and relatively low median scores were observed for VI. Notably, the four subscales were moderately correlated across dimensions of individualism–collectivism and horizontalism–verticalism (See Supplemental Figure 1). Furthermore, HI was positively correlated with conscientiousness, agreeableness, openness and emotional stability; VI was positively correlated with extraversion and negatively correlated with agreeableness, conscientiousness and openness; HC was correlated with agreeableness, conscientiousness, openness, emotional stability and extraversion; and VC was correlated with agreeableness, conscientiousness, emotional stability, extraversion and openness.

Table 3 shows results for multivariable regression models of each COVID-19 prevention variable, individually. These models controlled for demographic and lifestyle factors. As noted above, vaccination status was also controlled for in each of the behavioral prevention models due to its theoretical importance in shaping other prevention behaviors (i.e. perceived conferral of immunity). After controlling for these confounding factors:

- Vaccination was associated with higher extraversion (*p* = .023) and lower openness to experience (*p* = .001).
- Hand washing was associated with higher conscientiousness (*p* < .001).
- Mask wearing was associated with higher horizontal individualism (*p* = .008), higher agreeableness (*p* < .001), higher extraversion (*p* = .014) and lower emotional stability (*p* = .002).
- Reducing social interactions was associated with higher horizontal individualism (*p* = .015) and higher agreeableness (*p* < .001).
- Staying home (i.e. avoiding non-essential trips into community) was associated with higher agreeableness (*p* = .001) and higher vertical collectivism (*p* = .031)
- Neither physical distancing nor household bubbling were associated with any of the dimensions of culture or personality measured here (*p* > .05).

**Table 3.** Multivariable models testing associations with COVID-19 prevention behaviors.

	Fully or Partially Vaccinated (vs. Not)	Frequent Hand Washing	Mask Wearing in Public	Reducing Number of People Interacted With	Physical Distancing by 2 Meters from Others	Socializing Indoors Only with Household	Avoiding Non-Essential Trips in Community
	*aOR (95% CI)*	*aOR (95% CI)*	*aOR (95% CI)*	*aOR (95% CI)*	*aOR (95% CI)*	*aOR (95% CI)*	*aOR (95% CI)*
HC	0.98	1.00	1.03	0.98	1.02	1.00	0.98
	(0.93,1.02)	(0.96,1.04)	(0.99,1.07)	(0.94,1.02)	(0.98,1.05)	(0.97,1.04)	(0.94,1.01)
HI	0.97	1.02	**1.05**	**1.04**	1.01	1.02	1.03
	(0.94,1.01)	(0.98,1.06)	**(1.01,1.09)**	**(1.01,1.08)**	(0.98,1.04)	(0.99,1.05)	(0.99,1.06)
VC	1.03	1.03	1.00	1.03	1.02	1.00	**1.04**
	(0.99,1.07)	(0.99,1.07)	(0.96,1.04)	(0.99,1.06)	(0.99,1.06)	(0.97,1.03)	**(1.00,1.07)**
VI	1.00	1.00	0.98	0.98	0.99	1.02	1.01
	(0.96,1.03)	(0.96,1.04)	(0.95,1.02)	(0.95,1.01)	(0.96,1.02)	(0.99,1.05)	(0.98,1.04)
Agreeableness	1.05	1.09	**1.26**	**1.19**	1.06	1.09	**1.18**
	(0.94,1.18)	(0.98,1.21)	**(1.13,1.40)**	**(1.08,1.31)**	(0.96,1.16)	(0.99,1.19)	**(1.07,1.30)**
Conscientiousness	0.97	**1.18**	1.09	1.06	0.99	1.04	1.04
	(0.88,1.06)	**(1.08,1.29)**	(1.00,1.19)	(0.97,1.14)	(0.92,1.06)	(0.97,1.12)	(0.96,1.12)
Emotional Stability	0.99	1.05	**0.87**	0.97	1.02	0.99	1.00
	(0.91,1.08)	(0.96,1.13)	**(0.80,0.95)**	(0.90,1.04)	(0.95,1.09)	(0.92,1.06)	(0.93,1.07)
Extraversion	**1.10**	0.98	**1.12**	0.99	1.00	0.99	1.04
	**(1.01,1.19)**	(0.90,1.06)	**(1.02,1.22)**	(0.92,1.07)	(0.94,1.08)	(0.92,1.06)	(0.97,1.11)
Openness	**0.85**	1.04	0.95	1.05	1.05	0.97	0.95
	**(0.76,0.93)**	(0.94,1.15)	(0.86,1.05)	(0.96,1.15)	(0.97,1.14)	(0.89,1.05)	(0.88,1.03)
Tjur’s Pseudo-R^2^	0.240	0.113	0.139	0.167	0.113	0.082	0.100

Note: **BOLD** values indicate statistical significance at *p* = .05; aOR = Adjusted odds ratio, 95%CI = 95% Confidence Interval; HI = Horizontal Individualism, VI = Vertical Individualism; HC = Horizontal Collectivism, VC = Vertical Collectivism; All models controlled for confounding effects of age, gender, ethnicity, sexual orientation, educational attainment, student status, employment status, distance learning and working situation, income, province of residence and relationship status. With the exception of the vaccination model, all models also controlled for vaccination status.

**Figure 1.**
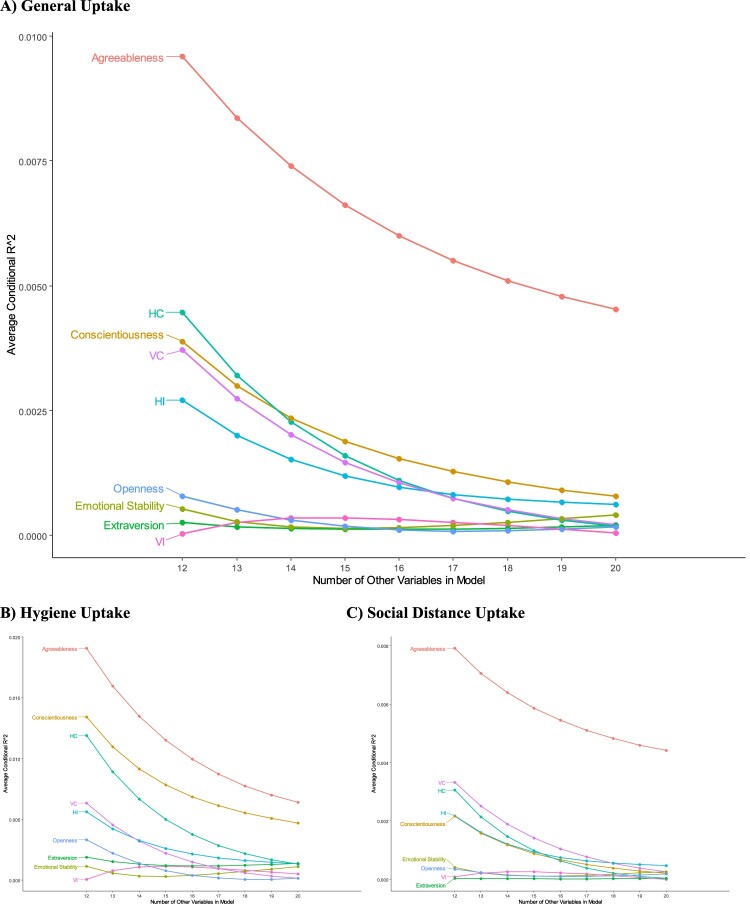
Dominance plots for COVID-19 prevention uptake indeces (A) general uptake, (B) hygiene uptake and (C) social distance uptake.

Figure 1 shows the results of the dominance analysis, which aimed to identify the most important model features predicting general uptake of the COVID-19 prevention measures. These analyses were based on an index counting the number of measures an individual was compliant to sub-indices measuring uptake of personal hygiene measures and social distancing measures were also analyzed. It is important to note that the index measures a slightly different concept than the individual multivariable models, which predict complying ‘very closely’ to each prevention behavior. The Index score captures a greater tendency towards compliance, with nuance provided for ‘sometimes’ engaging in the practice. In summary, these results show that, holding demographic and lifestyle factors constant:

- Agreeableness was the most important predictor for overall COVID-19 Prevention Behavior Index scores, followed by HC, Conscientiousness, VC and HI – which all had similarly moderate correlations with overall general uptake that were notably lower than the correlation with agreeableness. Openness, emotional stability, extraversion and VI all had consistently low average conditional contributions to R-squared, even when no additional factors were included in the model. As more of these personality and cultural variables were controlled for – the correlations with of HC, Conscientiousness, VC and HI became similarly small.
- For the personal hygiene sub-index, agreeableness was again the most important feature, followed by associations with conscientiousness and HC. The associations with VC, HI, openness, extraversion, emotional stability and VI were all small. As with the overall model, as more variables were controlled for, the independent contributions of measures declined, however agreeableness and conscientiousness remained notably elevated above other variables.
- For the social distance sub-index, agreeableness was again the most important. Similar, but elevated contributions from VC, HC, HI and conscientiousness were all observed, as were small contributions from emotional stability, openness, VI and extraversion. However, as with the general uptake measure, only the association with agreeableness remained notable.

**Figure 2.**
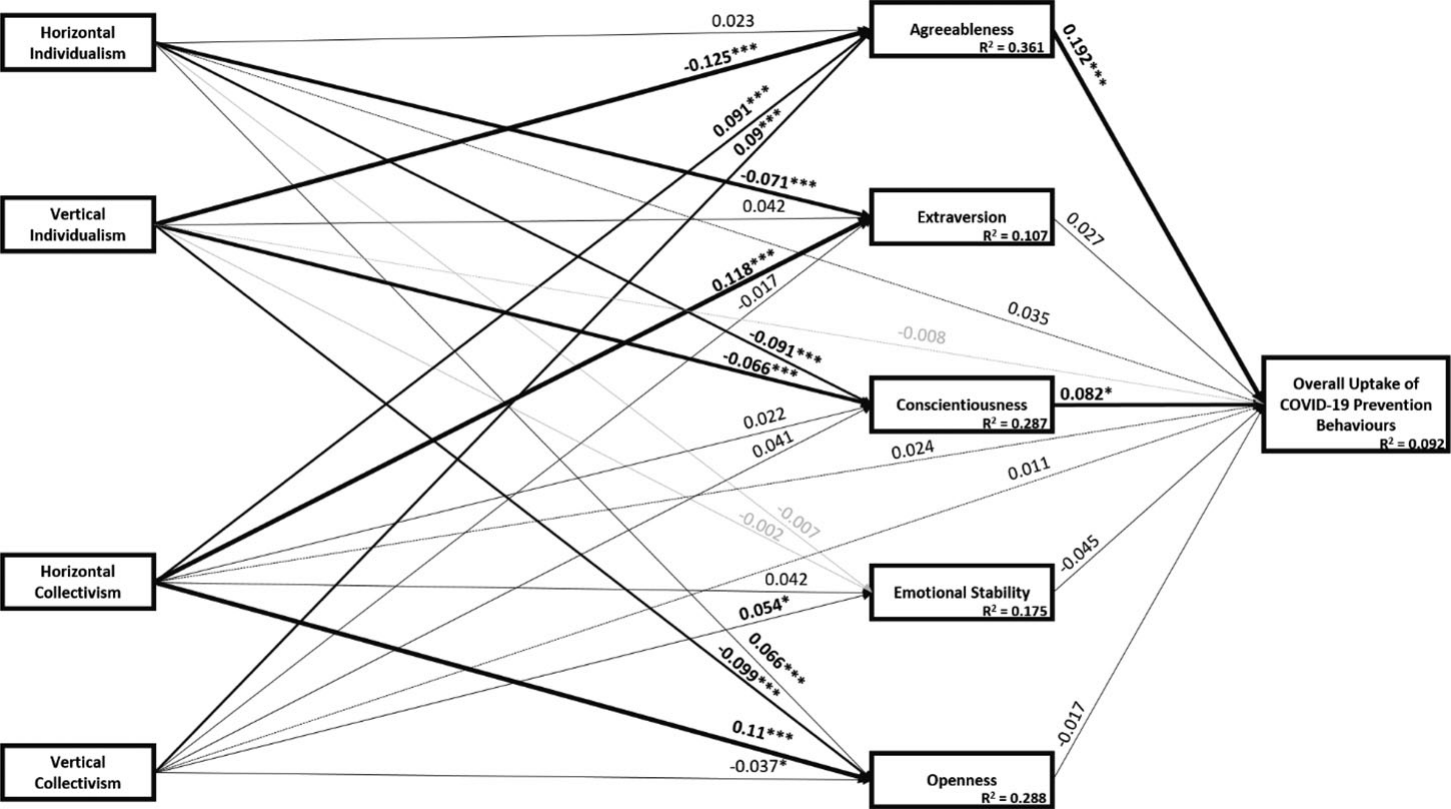
Structural equation model testing mediation of the relationship between cultural orientations and general uptake of COVID-19 prevention behaviors by personality factors.

To assess whether the relationships between HC, VC, HI and VI and overall COVID-19 Uptake Index scores were mediated by personality traits – particularly agreeableness – a post-hoc structural equation model was created, controlling for demographic confounders and including all five personality traits and each of the four cultural orientation subscales. Figure 2 shows results of these mediation analyses with the standardized regression estimates and *p*-values from the structural equation model displayed. In summary, overall COVID-19 Uptake Index scores were correlated with direct effects of agreeableness (B = 0.192 [0.068–0.283]) and conscientiousness (B = 0.082 [0.003–0.165]), but not extraversion (B = 0.027 [−0.03–0.084]), emotional stability (B = −0.045 [−0.153, 0.025]) or openness (B = −0.017 [−0.134, 0.082]). Similarly, the direct effects of HC (B = 0.024 [−0.013, 0.082]), VC (B = 0.011 [−0.044, 0.043]), HI (B = 0.035 [−0.01, 0.065]) and VI (B = −0.008 [−0.044, 0.029]) were not statistically related to the general COVID-19 Uptake Index scores. However, statistically significant indirect effects, operating through agreeableness, were statistically significant for VI (B = −0.024 [−0.037, −0.009]), HC (B = 0.017 [0.006, 0.030]) and VC (B = 0.017 [0.006, 0.033]), but not HI (B = 0.004 [−0.0003, 0.013]). No other indirect effects were observed through any of the other personality traits. The total effects of HI (B = 0.044 [0.005–0.078]), VI (B = −0.035 [−0.068–0.01]) and HC (B = 0.043 [0–0.093]) were significant, but not the total effect of VC (B = 0.03 [−0.017–0.06]) was not.

## Discussion

The present study sought to examine the independent correlations of cultural orientations and personality traits with the uptake of COVID-19 prevention behaviors among people living in Canada. In doing so, this exploratory study tested whether dimensions of both individualism and collectivism would be associated with higher uptake of COVID-19 prevention behaviors (Ludeke, Vitriol, Larsen, & Gensowski, 2021). This hypothesis was partially vindicated by several findings. First, it was observed that horizontal (egalitarian) individualism was associated with uptake of masking and reducing social interactions; while higher vertical (hierarchical) collectivism was associated with complying to stay-at-home guidelines. However, vaccination, hand washing, physical distancing and household bubbling were not associated with any of the four cultural orientations investigated. Surprisingly, neither horizontal collectivism nor vertical individualism were associated with any individual prevention behavior.

Yet, when examining the factors associated with the general uptake of COVID-19 prevention behaviors as an index measure, horizontal individualism and collectivism were both associated with greater uptake; while vertical individualism was associated with lower uptake. However, the direct effects for these cultural orientations were not significant and analyzing the indirect effects reveals that their operation is primarily through impacts on the psychological trait of agreeableness. As reviewed in the introduction section, the general finding that sub-dimensions of individualism and collectivism are associated with uptake of COVID-19 prevention behaviors is well supported in the context of previous studies using nuanced measures of collectivism and individualism (Galang et al., 2021). For example, Mo & Park demonstrated that the civic nature of horizontal individualism produces greater uptake of mask-wearing behavior (Mo & Park, 2021). Others have also found VI and HC to be significantly associated with COVID-19 related distress and uptake of prevention strategies, such as social distancing (Biddlestone, Green, & Douglas, 2020; Castle, Guilmi, & Stavrunova, 2021; Xiao, 2021). However, there are also some contradictions between the present research and previous studies. For example, Mo & Park reported that the obedient nature of vertical collectivism promoted COVID-19 compliance, whereas in the present study, there was no evidence for an effect of vertical collectivism. It is possible that differences with these other studies may be due to factors inherent to Canada or the period of data collection (i.e. Third Wave of COVID19). Alternatively, it may be that the inclusion of personality traits and other confounders mediated these relationships – demonstrating a potential strength of this exploratory study.

Indeed, the inclusion of personality measures allowed us to identify the independent and adjusted relationships between cultural orientations and COVID-19 prevention uptake. The present study’s models have accounted for at least five important psychological dimensions, plus a slue of demographic and lifestyle factors. These analyses showed that while each cultural dimension could be correlated with at least some dimension of COVID-19 prevention behavior, these relationships are mediated by other psychological factors. As more of these factors are controlled for, the associations with cultural orientation on behavior was seen to decline in our dominance analyses. The most notable mediator of the link between cultural orientations and COVID-19 prevention uptake was agreeableness. These findings not only help to better isolate the relationship between cultural orientations and behavior but they also provide insight into the relationship between culture and personality – namely that agreeableness may be a byproduct of cultural orientation.

Our study, of course, is not the first to consider the relationship between culture and personality. Existing studies suggest a considerable proportion (between 40 and 60%) of the Big Five personality traits are heritable (Jang, Livesley, & Vemon, 1996) and the remaining is socially influenced. It is widely accepted, that mean levels of the Big Five personality traits do vary between cultures – suggesting these personalities are responsive to cultural and social pressures within a given society (Kajonius, Mac Giolla, & Tran, 2017; McCrae & Terracciano, 2005; Schmitt et al., 2007; Triandis, 2001). The links between personality and culture identified in this exploratory study add empirical support to this understanding. Other researchers have noted that there are relatively few studies looking at links between collectivism and personality traits. The present study helps fill this gap, and the studies that do exist mostly agree with the findings herein. For example, Tychmanowicz, Filipiak and Sprynska reported that agreeableness was associated with less individualism and higher collectivism (Tychmanowicz, Filipiak, & Sprynska, 2021). Other researchers reporting similar findings have suggested that this association arises from a predisposition towards social dominance (e.g. competing, winning and asserting) among collectivists and a predisposition towards social submission (e.g. submitting, agreeing and going along) among collectivists (Moskowitz, Suh, & Desaulniers, 1994). Likewise, Realo, Allik and Vadi also found that collectivism is associated with greater agreeableness; adding conscientiousness to the list as well (Realo, Allik, & Vadi, 1997). While an agreement between these findings and ours is promising, it should be noted that the relationship between cultural orientations and personality traits likely varies from place to place (Tychmanowicz et al., 2021). For example, because individuals with collectivistic cultural orientations may be more strongly influenced by, or more sensitive to, their culture than individuals with individualistic orientations, the relationships between personality and culture may very across cultural orientations (Triandis, 2001).

In addition to describing the relationship between personality and culture, the present study’s findings also speak to the relative importance of personality traits in predicting COVID-19 prevention uptake. As noted, it is clear that agreeableness is a very important factor in predicting COVID-19 prevention uptake. The above noted relationship between collectivism and agreeableness thus helps frame why it might mediate the relationship between collectivism and COVID-19 prevention uptake – and helping us to understand why hypothesized links between COVID-19 prevention uptake and horizontal collectivism were not observed. Furthermore, it was observed that extraversion and openness to experience were associated with vaccine uptake – perhaps suggesting that introverted individuals may find it more difficult to navigate the health system in order to get the vaccination. Likewise, the positive association between vaccine uptake and openness to experience supports the notion that people who are less open (i.e. more hesitant) are less likely to uptake vaccines (Murphy et al., 2021). Addressing vaccine conspiracy theories and misinformation is therefore needed to support access to vaccines among these less open individuals. Findings also indicated that lower emotional stability might have the positive effect of encouraging mask uptake among relatively more neurotic individuals, who use masks to protect themselves from threats. However, it is unclear the extent to which mask wearing reduces anxieties (Saint & Moscovitch, 2021). Each of these findings speaks to the difficulty of achieving perfect uptake of COVID-19 prevention behaviors when individuals differ in their capacities and motivations to comply (De Carvalho, Pianowski, & Gonçalves, 2020). Carefully planned rollout and promotion of COVID-19 prevention guidelines could thus support individuals who may experience unique, person-level barriers to COVID-19 prevention uptake. For example, emphasizing personal AND collective benefits, highlighting the normative nature of prevention and disseminating these messages through trusted social sources could improve the cultural and social diffusion of health promotion messages. Of course, crafting these interventions will require additional research, particularly to understand how disagreeableness can be overcome in health promotion strategies.

## Limitations

Despite several contributions to the literature, this exploratory study has limitations. First, the ways individuals react to the pandemic may change over time (Wright & Fancourt, 2021). The present cross-sectional analysis is limited to a snapshot in time – conveniently just following the peak of the third wave of the COVID-19 pandemic. It is possible that different relationships between cultural orientations, personality traits and COVID-19 prevention behaviors might shift over the course of the pandemic – especially as people become tired of these practices. The study from which these data are drawn is designed with a longitudinal sub-cohort that will eventually support annual data collection. This and other studies will allow for future analyses to account for temporal changes in these relationships. Second, the sampling method used in the present study is vulnerable to several biases. Important differences between people who opt in to complete surveys and those who do not is one such source of bias. Likewise, the use of online recruitment, while necessary due to the COVID-19 pandemic, does limit the sampling frame to people engaged on social media sites. While ads ran for several weeks, increasing the chance of recruiting infrequent users, it is hard to adequately account for these potential selection biases. One potential strength of this sampling design was reaching a demographically diverse population. Nevertheless, replication using other sampling methods is needed. Further, as this survey was designed to address several research questions, the survey was long (taking approximately 21 minutes to complete, on average). As such, some participants dropped out and did not finish the survey or were excluded from this analysis due to missing data. Missing data due to non-completion may be another importance source of bias – particularly as non-response could be related to personality traits. While steps were taken to reduce participant burden (e.g. randomizing some sections of the survey), a shorter survey design might allow for validation of study results with a lower drop out rate than observed here (∼30%). In comparing the initial sample size to the analytic sample size, it should be noted that only some participants in the full survey were eligible for inclusion – mainly because individuals were randomized to complete some sections but not others in order to reduce participant burden. While these survey design strategies, along with participant incentives, hopefully minimized bias from non-response and drop out, it is likely that a shorter survey, with better compensation, could result in better response rates. Finally, this exploratory study is limited by imperfect measures (e.g. TIPI, Cultural Orientations Scale and COVID-19 Prevention Uptake Index). While the measures appear to have evidence supporting their validity, it would be appropriate to replicate the findings with other scales that are carefully selected to measure the hypothesized pathways explored in the present study.

## Conclusion

In conclusion, this exploratory study illustrates that both collectivism and individualism are associated with distinct patterns of COVID-19 prevention practice. Further, this exploratory study shows that the relationships between these cultural orientations and COVID-19 prevention behavior is partially mediated by personal characteristics and situational factors. Notably, agreeableness appears to be a key factor promoting uptake of COVID-19 prevention behaviors, and it is therefore important to understand how to promote public health messages to disagreeable and culturally-diverse communities.
